# Performance of Mali’s Biosafety Level 3 Laboratory in the External Quality Assessment in Preparedness of Laboratory Accreditation and Support to Clinical Trials

**DOI:** 10.4103/ijmy.ijmy_5_20

**Published:** 2020

**Authors:** N Coulibaly, B Kone, M Sanogo, ACG Togo, B Diarra, YS Sarro, AB Cisse, O Kodio, G Coulibaly, M Kone, B Baya, M Maiga, D Dabitao, M Belson, S Dao, S Diallo, M Diakite, AH Babana, S Doumbia

**Affiliations:** 1 University Clinical Research Center-SEREFO-Laboratory, University of Sciences, Techniques and Technologies of Bamako, Mali; 2 National Referral Laboratory of Mycobacteriology, National Institute of Public Health, INSP, Bamako, Mali; 3 Center for Global Health, Northwestern University, Chicago, IL; 4 Collaborative Clinical Research Branch, Division of Clinical Research, National Institute of Allergy and Infectious Diseases, National Institutes of Health, Bethesda, Maryland, USA; 5 Microbiology and Biotechnology Research Laboratory, Faculty of Sciences and Techniques, University of Sciences, Techniques and Technologies of Bamako, Bamako, Mali

**Keywords:** External quality assessment, Mali, mycobacteriology

## Abstract

**Background:**

The external quality assessment (EQA) or external quality control is an evaluation conducted by a certified external organization to inquire about the quality of the results provided by a laboratory. The primary role of EQA is to verify the accuracy of laboratory results. This is essential in research because research data should be published in international peer-reviewed journals, and laboratory results must be repeatable. In 2007, the University Clinical Research Center (UCRC’s) biosafety level 3 (BSL-3) laboratory joined the EQA program with the College of American Pathologists in acid-fast staining and culture and identification of mycobacteria as per laboratory accreditation preparedness. Thus, after 11 years of participation, the goal of our study was to evaluate the performance of our laboratory during the different interlaboratory surveys.

**Methods:**

We conducted a descriptive retrospective study to evaluate the results of UCRC mycobacteriology laboratory from surveys conducted during 2007 and 2017.

**Results:**

Of the 22 evaluations, the laboratory had satisfactory (100% of concordance results) in 18 (81.8%) and good (80% of concordance results) in 4 (18.2%). Overall, the laboratory was above the commended/accepted limits of 75%.

**Conclusion:**

So far, UCRC’s BSL-3 performed well during the first 11 years of survey participation, and efforts should be deployed to maintain this high quality in the preparedness for laboratory accreditation and support to clinical trials.

## Introduction

The external quality assessment (EQA), previously known as external quality control, is a verification by a certified external organism of the quality of the results provided by a laboratory on samples known to the external organism but unknown to the laboratory.^[1]^ It is a retrospective control allowing interlaboratory confrontation in order to improve the quality of the work of all the participants.^[1]^

The College of American Pathologists (CAP) is a world leader proficiency testing and accreditation organism with more than 20,000 participating laboratories around the world.^[2]^ From routine to more specialized samples, the program helps laboratories to stay at high-quality performance and accuracy in patient results.

The EQA program in microbiology was initiated by the World Health Organization (WHO) in July 2002 with the support of the United States Agency for International Development and the Global Vaccine Alliance.^[2]^ The Office of the WHO for the Africa Region, in collaboration with the National Institute for Communicable Diseases, set up this program to assess national laboratories’ capacity to implement standardized methods, to improve their capacity for surveillance and response adapted to priority diseases. In 2005, malaria and tuberculosis (TB) became part of the EQA.^[3]^

TB remains a major public health problem with 10 million cases, 1.3 million of death around the world in 2018. The emergence of the human immunodeficiency virus (HIV) and anti-TB-resistant strains (480,000 multidrug-resistant cases) increases the threat.^[4]^ It is a contagious disease caused by mycobacteria belonging to the *Mycobacterium tuberculosis* complex (MTBc) family.^[4]^ Paradoxically, TB remains underdiagnosed and untreated in resource-limited setting, with only 57% of detection rate in Mali in 2017.^[4]^ Its control aims to reduce the spread of infection^[5,6]^ by early diagnosing and treating pulmonary cases which are more infectious. Indeed, between 2000 and 2015, early diagnosis and adequate treatment of TB saved 49 million lives.^[7]^ However, the diagnosis of TB is essentially based on smear microscopy.^[8]^ The good quality of sputum smear remains one of the important components of the Directly Observed Treatment, Short-Course strategy which is highly recommended by the WHO for TB control. Laboratory results of a suspected patient are used by physicians to initiate or refute anti-TB treatment. In addition, as recommended in the WHO guidelines, treatment success monitoring is also followed by sputum smear examination using Ziehl–Neelsen or auramine (A/R) fluorescent microscopy (FM), in which conversion from smear positive to smear negative at 2 months is an important predictor of treatment success.^[9,10]^ Therefore, the laboratory becomes crucial in diagnosing and monitoring TB patients.

In Mali, EQA for clinical sample is very limited, and only TB program is trying to implement it. There is no national program for EQA.^[11]^

Since its creation in 2006, University Clinical Research Center (UCRC) biosafety level-3 (BSL-3), previously called SEREFO (HIV/TB Research and Training Center) laboratory program, has set up an EQA system with CAP. Thus, the purpose of this study was to evaluate the performance of UCRC BSL-3 TB laboratory after 11 years of participation in CAP microbiology proficiency testing.

## Materials and Methods

### Study setting

The UCRC is a clinical research program created under the collaboration between Mali and the USA through their ministries of health. The UCRC’s mission is to continuously improve the quality of health care nationally, regionally, and globally by facilitating excellent clinical research at international standards, strengthening research capacity, and providing training.^[11]^ One of its composed laboratories is the BSL-3 where TB testing is conducted safely.

### Study design

This was a cross-sectional study. The data were collected from BSL-3 Laboratory CAP’s evaluation reports between 2007 and 2017.

### Sample types and shipping to Bamako site

Samples received in the laboratory for analysis come from the CAP. These samples are usually a reconstituted human tissue, and the companies guarantee the quality, homogeneity, and stability of the samples. A total of five samples were well packed and marked with the infectious substances label before shipping to all laboratories participating in the survey (sites). The EQA for mycobacterial (Acid-Fast Staining and Mycobacterial Identification: Mycobacteriology Limited: E1-B Program under CAP) test is sent to the laboratory twice a year for testing. Each laboratory participating in the survey will perform 10 samples per year. Samples are coded so that the laboratory technician is blinded to the diagnosis by including undifferentiated positive and negative samples in the survey. In the laboratory, the samples should be performed like routine sample arriving for TB diagnosis in the laboratory by following standard operating procedures. The results are then reported on the CAP website using the codes on the tubes. The CAP assesses the results and scores from 0% to 100% of correct answer. The laboratory performance is considered acceptable when the survey scores are >75%.^[2]^

### Laboratory methods for culture and identification

Specimen samples were digested and decontaminated using the standard N-acetyl-L-cysteine/4% NaOH solution, concentrated by centrifugation (4500 rpm), and inoculated on both liquid (*Mycobacterium* Growth Incubator Tube [BBL^™^ MGIT^™^ Becton Dickinson, Sparks, MD, USA]), and solid (Middlebrook 7H11 Agar and Selective 7H11 Agar) media. Simultaneously, an aliquot of concentrated specimen was prepared for indirect commercial A/R staining (BBL^™^ Becton Dickinson, Sparks, MD, USA). Speciation of positive mycobacterial cultures was based on acid-fast bacilli (AFB) positivity in FM and colony morphology on solid medium and was confirmed by nucleic acid probes (AccuProbe® Gen-Probe, San Diego, CA, USA), or Capilia^™^ TB-Neo assay, TAUNS Laboratories, Inc., Futaba-Cho, Numazu, Shizuoka, Japan.

### Fluorescent auramine staining protocol and grading of smear microscopy

A/R staining protocol is a very old staining technic used in the TB diagnosis and its treatment follow-up. The property of acid-fastness is based on the presence of mycolic acids in the mycobacterial cell wall. The primary stain (A/R) binds strongly to the mycolic acids of the cell wall. Intense decolorization (strong acids, alcohol) does not release primary stain from the cell wall and the mycobacterial retain the fluorescent bright yellow color of A/R. Potassium permanganate or TB methylene blue is used to quench the fluorescence in the background.

The grading of sputum smear microscopy was done following the International Union against Tuberculosis and Lung Diseases grading criteria as negative (no AFB seen or 0), few AFB seen (10–99 AFB seen in 100 fields or 1+), moderate AFB seen (1–10 AFB seen per field or 2+), and many AFB seen (>10 AFB seen per field or 3+).^[12]^ Following these tests, the results were reported on the CAP website for evaluation.

### Statistical analysis

The data were collected from the result sheets (CAP Evaluation report or Annex-4) sending to the laboratory by CAP program and entered into the Excel sheet. The data were analyzed using the SPSS Software version 21.0 (IBM SPSS, Armonk, New York: IBM Corporation) to evaluate laboratory performance.

## Results

### Number of samples received during the 11 years of survey participation

**Figure 1:**
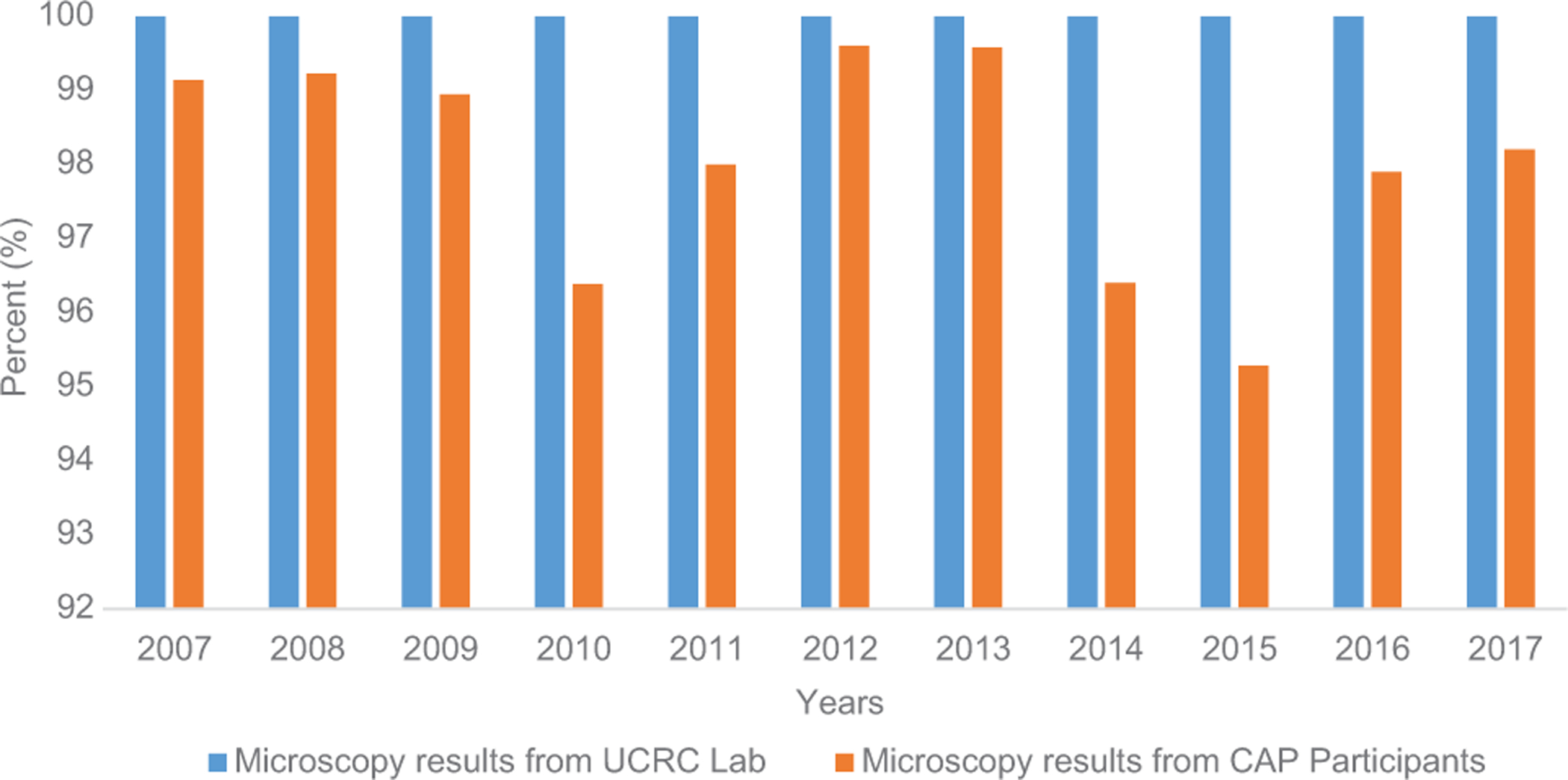
Comparison of University Clinical Research Center biosafety level-3 laboratory score to the score of other laboratories as for microscopy reading

In total, 90 sample tests in 22 surveys were received for EQA between 2007 and 2017 in UCRC’s BSL-3 laboratory [Table 1]. Overall, we obtained satisfactory (100% of concordance) results in 18 (81.8%) surveys and good (80% of concordance) results in 4 (18.2%) surveys [Figure 1].

### Smear and culture results

**Figure 2:**
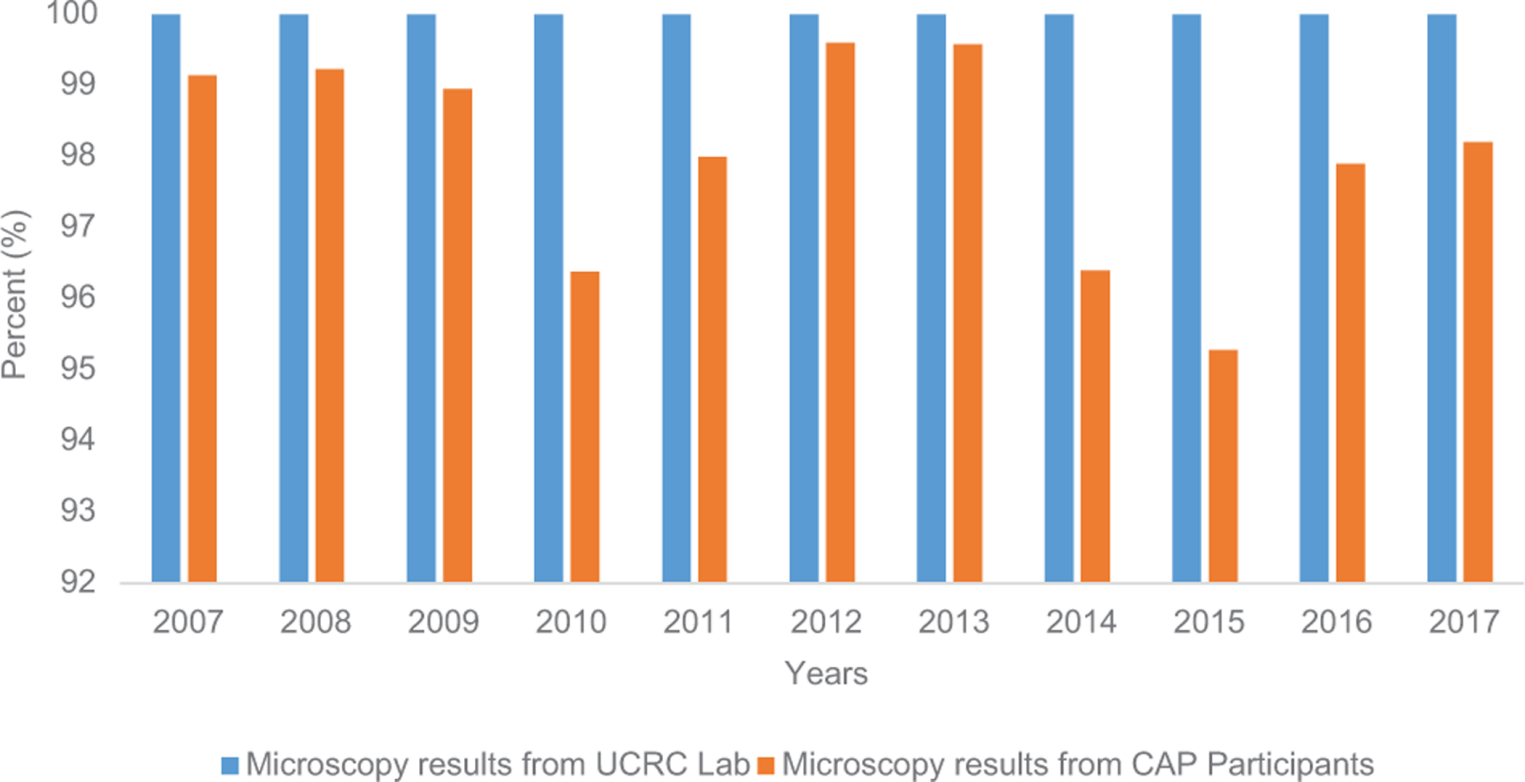
University Clinical Research Center biosafety level-3 laboratory score compared to the score of the other laboratories according to the culture technique

**Figure 3:**
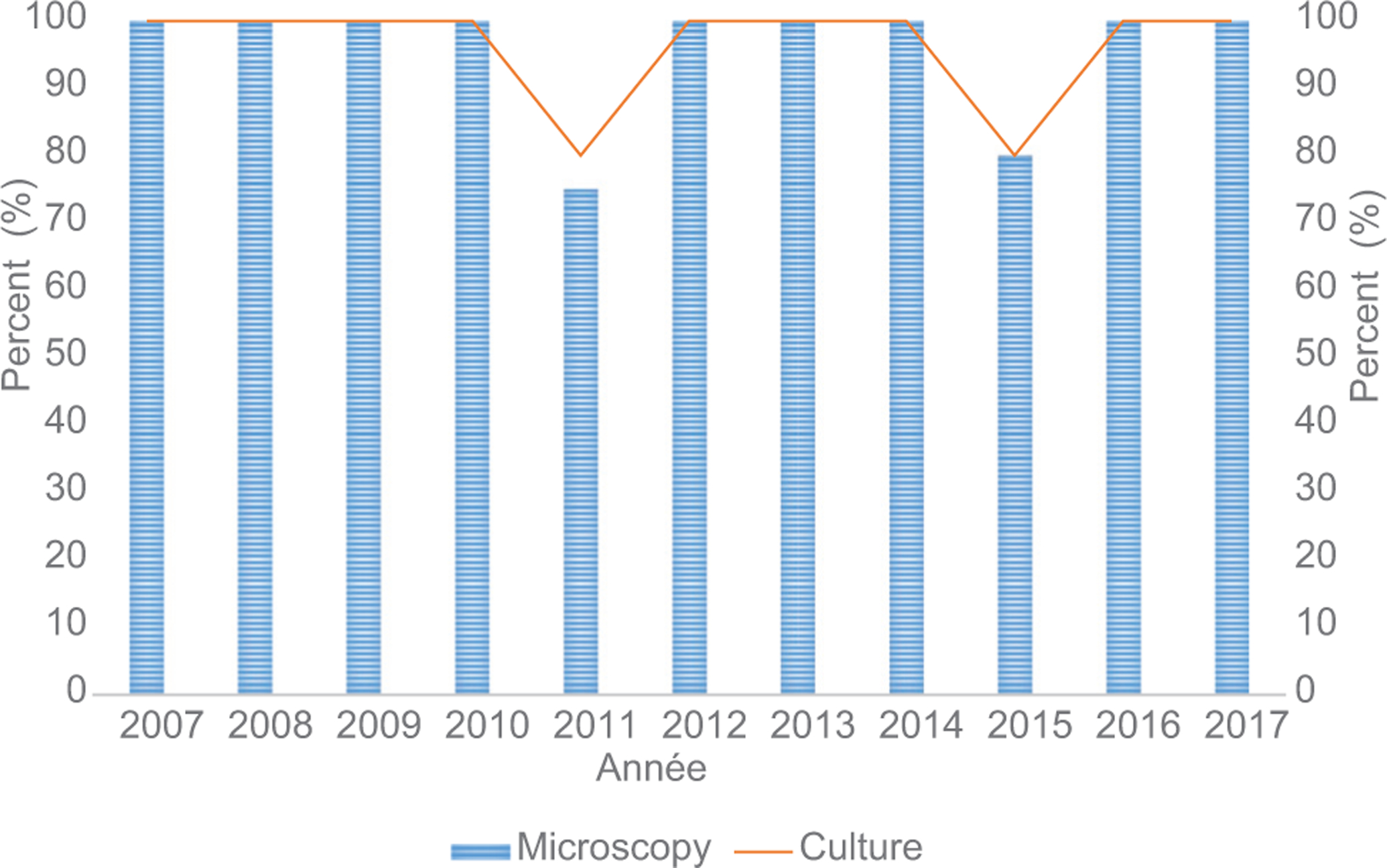
Performance of University Clinical Research Center biosafety level-3 laboratory in the external quality assessment between 2007 and 2017

Between 2007 and 2017, a total of 529 laboratories participated to the CAP’s EQA survey for smear microscopy for AFB identification. Except the years 2011 and 2015, our results were 100% of concordance with expected results and above the global average of other participating laboratories [Figures 2 and 3].

During the same period, 480 laboratories performing culture and identification of mycobacteria were participating in the survey. Our score was 100% in all the 22 surveys except for the years 2011 and 2015 [Figures 2 and 3].

### Performance of University Clinical Research Center personnel

During the 2-year period 2016 and 2017, we evaluated the performance of our personnel, and we had 100% of sensitivity, specificity, positive, and negative predictive values in smear microscopy and culture of mycobacteria [Table 2].

## Discussion

EQA is an essential component of quality control and is an effective way to identify problems and verify laboratory performance against other laboratories using external agencies.^[13–15]^ In this study, we assessed the overall performance of UCRC BSL-3 after 11 years of proficiency testing under CAP. We found that our laboratory results in smear microscopy and culture of mycobacteria are in good agreement and have good sensitivity and specificity with CAP’s expected results [Table 2]. Therefore, we demonstrate the continuous efficacy and efficiency of the personnel.^[16]^ EQA programs are increasingly needed and essential for increasing confidence in the laboratory results that must be used in the diagnosis, treatment, or epidemiological surveillance of certain diseases.^[17,18]^ Moreover, it appears necessary for each Biomedical Analysis Laboratory (BAL) to be enrolled in EQA programs in order to improve their services and their result reliability. Research laboratory also requires this kind of EQA for their research or clinical trial result validation.^[19–23]^

Given the pivotal role of accurate diagnosis of TB by direct examination, culture and molecular techniques,^[24]^ and mainly in resource-limited countries with an increased number of underdiagnosed patients,^[25]^ strategies to initiate EQA implementation should be encouraged in these regions even only at one laboratory. An example is Mali, where the north of the country suffers from instability due to terrorist groups, despite the presence of peacekeeping forces since 2013. Thus, TB diagnostic services in such a setting are especially challenging, as no supervision visits by the national TB program can be conducted to the north of the country. Therefore we cannot guarantee good results from those laboratories. In our study, the number of participating laboratories was 529 for the microscopy survey and 480 for the culture. This implies that several laboratories are participating in CAP program and confirmed that our results were more discussed and valued. With smear results, we had 100% agreement of the laboratory results with those expected from CAP during a period of 8 years out of 11 years of participation, thus demonstrating the accuracy of our results. These results are identical to that of Ssengooba *et al*. in Uganda in 2015.^[26]^ However, the concordance results during the years 2011 and 2015 were 75% and 80%, respectively. These results could be explained by the hiring of new technicians in the laboratory in 2011 followed by a poor orientation training and by the burden of laboratory work in 2015 with the advent of the Ebola virus disease (EVD) epidemic in our country.^[27]^

During the EVD epidemic, in addition to suspected Ebola sample testing, the team was continuing working on TB samples. However, the scores were still within the allowed limit which is 75% of concordance.^[28]^ The culture is the gold standard in TB diagnosis because it is based on the capacity of bacilli to grow on specific culture media and to demonstrate their viability. We had the same performance in smear and culture.

High sensitivity and specificity are highly recommended for all diagnostic tests mainly for TB. In our study, we obtained 100% of concordance in 8 years demonstrating the efficacy and efficiency of the laboratory for the identification of MTBc. Due to laboratory certification and maintenance of the BSL-3, we missed one complete year of participation. Thus, we should identify alternative backup in such a situation. The high scores obtained by the laboratory during the 11 years were attributable to the continuous training and skill acquired over the years. UCRC leadership should continue monitoring the training record of the personnel so that they maintain and try to upgrade the gap in this record in order for the laboratory to maintain high quality. Moreover, the laboratory application for accreditation will be the most important step to follow.

Our desire to implement high-quality standardized laboratory results obliged us to register and participate in the WHO survey since 2018. These are 20 unknown samples prepared and shipped from the Institute of Tropical Medicine, Antwerp, Belgium, for culture and identification of mycobacteria and drug susceptibility testing (DST). So far, our results are in good concordance with expected results. This is crucial in this era of antimycobacterial drug resistance. In addition, the WHO portfolio will complete the capacity of a resource-limited TB laboratory with smear microscopy, culture, identification, and DST which are the most tests required with regard to laboratory accreditation and clinical trials.

The strengths of our study include the continuous performance over the 11-year period despite the movement of personnel and new tests within the laboratory. Thus, we were able to maintain a standard quality control test for a long period of time, in addition to our regular protocol works.

Our study has some limitations. First, we were not able to perform all the samples received due to laboratory shutdown for certification, but we failed also in identify some samples. Second, this is just smear, culture, and identification, and it will be interesting to have good performance in DST with CAP and WHO.

## Conclusion

UCRC BSL-3 mycobacteriology laboratory performed very well over 11-year participation in CAP EQA for smear and culture and must continue as well. This performance is of great importance to maintain high-quality work in diagnosing TB. These results should be used as proof to initiate the accreditation of the laboratory and to initiate clinical trials.

## Figures and Tables

**Table 1:** Overall participation of the University Clinical Research Center in the different surveys between 2007 and 2017 with the corresponding number of samples received and tested

Years	First survey	Second survey
2007	5 samples	5 samples
2008	5 samples	5 samples
2009	5 samples	5 samples
2010	5 samples	5 samples
2011	5 samples	0 sample
2012	5 samples	5 samples
2013	5 samples	5 samples
2014	5 samples	5 samples
2015	5 samples	0 sample
2016	5 samples	5 samples
2017	5 samples	5 samples

In 2011 and 2015, we missed the second shipment of the samples

**Table 2:** Performance of the trainer compares to that of a trainee at the microscopy and culture for the identification of *Mycobacterium tuberculosis* complex in 2016 and 2017

Trainee (UCRC results)	Trainer (CAP, expected results)			
	Year 2016		Year 2017	
	Microscopy	Culture	Microscopy	Culture
Sensibility (%)	100 (43.85–100)	100 (43.85–100)	100 (43.85–100)	100 (43.85–100)
Specificity (%)	100 (34.24–100)	100 (34.24–100)	100 (34.24–100)	100 (34.24–100)
Positive Predictive value (%)	100 (43.85–100)	100 (43.85–100)	100 (43.85–100)	100 (43.85–100)
Negative predictive value (%)	100 (34.24–100)	100 (34.24–100)	100 (34.24–100)	100 (34.24–100)
Precison of the test (%)	100 (56.55–100)	100 (56.55–100)	100 (56.55–100)	100 (56.55–100)

CAP: College of American Pathologists, UCRC: University Clinical Research Center, PPV: Positive predictive value, NPV: Negative predictive value, MTBc: *Mycobacterium tuberculosis* complex
